# Enterocins Produced by Enterococci Isolated from Breast-Fed Infants: Antilisterial Potential

**DOI:** 10.3390/children11020261

**Published:** 2024-02-17

**Authors:** José María Landete, Raquel Montiel, Eva Rodríguez-Mínguez, Juan L. Arqués

**Affiliations:** Department of Food Technology, Instituto Nacional de Investigación y Tecnología Agraria y Alimentaria, INIA-CSIC, Carretera de La Coruña Km 7, 28040 Madrid, Spain; landete.josem@inia.csic.es (J.M.L.); rakelm@inia.csic.es (R.M.); minguez@inia.csic.es (E.R.-M.)

**Keywords:** enterocins, *Enterococcus*, breast-fed infants, bacterial infection, *Listeria monocytogenes*, selective inhibition, probiotic

## Abstract

Enterocins are bacteriocins synthesized by *Enterococcus* strains that show an interesting antimicrobial effectiveness against foodborne pathogens such as *Listeria monocytogenes*. The objectives of this study were to identify and analyze the expression of enterocin genes of *Enterococcus* isolated from breast-fed infants and evaluate their ability to inhibit three human isolates of virulent *L. monocytogenes*, as well as some probiotic bacteria. The susceptibility of the strains of *L. monocytogenes* to fifteen antibiotics was tested, detecting their resistance to cefoxitin (constitutively resistant), oxacillin, and clindamycin. The production of enterocins A, B, and P was observed in *Enterococcus faecium* isolates, while enterocin AS-48 was detected in an *Enterococcus faecalis* isolate. AS-48 showed antilisterial activity by itself, while the joint action of enterocins A and B or B and P was necessary for inhibiting *L. monocytogenes*, demonstrating the synergistic effect of those combinations. The presence of multiple enterocin genes does not assure the inhibition of *L. monocytogenes* strains. However, the expression of multiple enterocin genes showed a good correlation with the inhibition capacity of these strains. Furthermore, the potential beneficial strains of lactobacilli and bifidobacteria examined were not inhibited by any of the enterocins produced individually or in combination, with the exception of *Bifidobacterium longum* BB536, which was inhibited by enterocin AS-48 and the joint production of enterocins A and B or B and P. The enterocins studied here could be candidates for developing alternative treatments against antibiotic-resistant bacterial infections. Moreover, these selected enterocin-producing *E. faecium* strains isolated from breast-fed infants could be used as probiotic strains due to their antilisterial effect, as well as the absence of virulence factors.

## 1. Introduction

The current worldwide increase in resistant bacteria has led to a search for novel antimicrobial agents. Within this scenario, bacteriocins and bacteriocin-like inhibitory compounds produced by lactic acid bacteria are promising tools against pathogenic bacteria due to the fact that they have been used safely for many years, and thanks to their stability in a wide range of physicochemical conditions, their mechanisms of action (which are different from the commonly used therapeutic products), and their ability to influence the host immune responses (acting as signaling peptides). Moreover, they can be subjected to gene-based peptide engineering [1].

*Listeria monocytogenes* causes listeriosis and is predominantly foodborne, particularly in ready-to-eat food. It most often affects vulnerable population groups, such as pregnant women, newborn infants, children, immunocompromised subjects, and the elderly. However, human listeriosis cannot always be attributed to the consumption of contaminated food products, having several potential methods of transmission, including neonatal cross-infection [2]. Antibiotics are the most common treatment choice for listeriosis, although antibiotic resistance in *L. monocytogenes* has been reported over the past few decades [3]. Listeriosis is an emerging infection representing a public health concern worldwide and has a high incidence in pregnant women [4,5,6,7,8,9]. Infection during pregnancy can cause fetal loss, premature birth, and illness or death in newborns [10].

*Enterococcus* spp. have been isolated from different environments and are indigenous inhabitants of the healthy human gastrointestinal tract. Some of them are able to produce ribosomally synthesized antimicrobial peptides or bacteriocins called enterocins. They are considered one of the most significant bacteriocin producers, both in number of bacteriocin-producing isolates and variety [11]. Enterocins are peptides secreted extracellularly during the metabolic process. Enterocins can be categorized into four major classes: Class I, known as lantibiotic enterocins; Class II, composed of small heat-stable, non-lantibiotic enterocins; Class III, or cyclic enterocins; and Class IV, which includes large peptides linked to lipids or carbohydrates. The increasing interest in bacteriocins produced by LAB has been enhanced by their spectrum of activity, being able to inhibit Gram-positive pathogens such as *L. monocytogenes*, *Staphylococcus aureus*, *Bacillus cereus,* or *Clostridium botulinum* [12]. *Enterococcus faecalis* and *Enterococcus faecium* are the species most commonly found in the human gut [13]. Due to their function in supporting well-being and health as part of the intestinal microbiota, the development of strains of enterococci as probiotics with beneficial effects on the health of the host has gained increasing interest [14]. However, the use of enterococci as a probiotic is controversial due to their role as opportunistic pathogens outside of the gastrointestinal tract [15]. Some studies suggest that cases of nosocomial infections by the genus only occur in vulnerable populations, including hospitalized children, and are caused by enterococci that contain virulence factors, especially vancomycin-resistant enterococci [16].

Breast-feeding can be a considerable source of enterococci for the nursing infant’s gut [17] and may have an influence on the overall composition of the microbiota and, therefore, on its physiological functions. In a previous work from our group, a total of 41 enterococci (26 *E. faecalis* and 15 *E. faecium*) were isolated from healthy breast-fed infants younger than 6 months, and the presence of virulence factors, antibiotic resistances, and biogenic amine production was evaluated [18]. Here, the evaluation of enterocin production by those enterococci was carried out, which can be of interest as alternatives to antibiotics due to the increase in the number of resistances both in clinical and commensal bacteria [19,20,21]. Several studies have been published in recent decades reporting that bacteriocins may be used as alternatives to antibiotics in the prophylaxis or treatment of bacterial infections, including those caused by multidrug-resistant (MDR) strains [22]. The use of bacteriocins as therapeutic agents, and as part of a multiple-hurdle approach with antibiotics, has been explored as a viable alternative that could also reduce the undesirable effects of antibiotics on the intestinal microbiota during the treatment of intestinal infections [23,24]. Moreover, the fact that *Enterococcus* strains have been isolated from infant feces could be an advantage over other strains isolated from foods [25,26] for their potential use as probiotics [11,27], since they would be able to compete with pathogenic bacteria for nutrients and establish the gastrointestinal tract [28].

The aim of this study was to analyze the occurrence of bacteriocin activity among enterococci isolated from breast-fed infants and their activity against virulent *L. monocytogenes* and beneficial bacteria. Additionally, purified or partially purified enterocins, as well as bacteriocinogenic enterococci strains, as alternative treatments against antibiotic-resistant *L. monocytogenes* infections were discussed.

## 2. Materials and Methods

### 2.1. Bacterial Cultures and Media

A total of 26 *E. faecalis* and 15 *E. faecium* isolates from fecal samples from 23 healthy breast-fed infants younger than 6 months were used in this study [18]. Isolates were cultured in MRS broth (Scharlau Chemie SA, Barcelona, Spain) at 37 °C in aerobic conditions.

*L. monocytogenes* strains were grown in TSB (Scharlau Chemie SA, Barcelona, Spain) at 37 °C in aerobic conditions. Lactobacilli strains were grown in MRS at 37 °C in aerobic conditions, whereas *Bifodobacterium* strains were grown in RCM broth (BD, Le Pont de Claix, Grenoble, France) at 37 °C in anaerobic conditions in sealed jars using AnaeroGen sachets (Oxoid, Ltd., Basingstoke, UK).

### 2.2. L. monocytogenes Antibiotic Susceptibility Testing

Antimicrobial susceptibility testing of the human isolates of virulent *L. monocytogenes* was performed on Müller–Hinton agar (Condalab, Madrid, Spain) supplemented with 5% defibrinated horse blood (Thermo-Fisher Scientific, Waltham, MA, USA) and 20 mg/mL β-NAD (Sigma-Aldrich, St. Louis, MO, USA) according to the disk diffusion method recommended by the European Committee on Antimicrobial Susceptibility Testing (EUCAST, Basel, Switzerland) [29]. The following antibiotics, chosen for using in the veterinary and human medicine for treatment of listeriosis, were tested: penicillin G (10 IU/disc), ampicillin (10 µg/disc), amoxicillin/clavulanic acid (30 µg/disc), oxacillin (1 µg/disc), gentamicin (10 µg/disc), chloramphenicol (30 µg/disc), vancomycin (30 µg/disc), tetracyclin (30 µg/disc), ciprofloxacin (5 µg/disc), clindamycin (2 µg/disc), erythromycin (15 µg/disc), cefoxitin (30 µg/disc), trimethoprim-sulphamethoxazole (25 µg/disc), meropenem (10 µg/disc), and rifampicin (5 µg/disc) (Oxoid). The control strain used in this study was *Staphylococcus aureus* ATCC 29213.

### 2.3. Detection of Enterocin Genes

Enterocin-encoding genes in the enterococci above mentioned were detected by PCR using the specific oligonucleotide primers listed in Table 1. The PCR were sequenced and compared with known sequences in the BLASTN database (National Center for Biotechnology Information—NCBI, Bethesda, MD, USA).

### 2.4. Bacteriocin Bioassay and Spectrum of Activity

The antimicrobial activity of enterococcal strains harboring enterocin genes was tested using *L. monocytogenes* Scott A, *L. monocytogenes* OHIO, and *L. monocytogenes* ATCC 19115 as indicator strains. Enterocin extracts were obtained by centrifuging at 10,000× *g* for 15 min at 4 °C an 18 h culture of the enterocin producer, followed by filtering the supernatant through a 0.22 mm pore size low protein binding filter (Millex GV, Millipore, Molsheim, France), and raising the pH to 6.5 with 2 M NaOH. Enterocin extracts were aliquoted and stored at −70 °C until use. Bacteriocin activities were evaluated using a microtiter plate assay as described by Holo et al. (1991) [37]. After aerobic incubation at 37 °C for 24 h, the growth of indicator strains was examined. The titer (expressed in enterocin units [EU] per milliliter) was defined as the reciprocal of the highest dilution that did not allow the growth of the indicator strain. Two independent trials were performed and each strain was assayed in duplicate.

Later, enterocin assays were also carried out against Lacticaseibacillus rhamnosus GG, Limosilactobacillus reuteri Biogaia, Limosilactobacillus reuteri INIA P572, Lacticaseibacillus paracasei INIA P272, Lacticaseibacillus rhamnosus INIA P344, Bifidobacterium longum BB536, Bifidobacterium animalis BB12, Bifidobacterium pseudolongum INIA P2, and Bifidobacterium breve INIA P18. Plates of MRS, RCM, or TSA were used for the different strains.

### 2.5. Total RNA Isolation and Reverse Transcription (RT)-PCR Analysis

The *Enterococcus* that harbor enterocin genes were grown in MRS broth (10 mL) overnight under aerobic conditions at 37 °C. The total RNA was isolated using the High Pure RNA isolation kit (Roche, Mannheim, Germany) as specified by the manufacturer. The RNA was quantified by measuring its optical density at 260 nm. The total RNA quality was assessed spectrophotometrically and with gel electrophoresis. Using a GeneAmpR EZ r*Tth* RNA PCR kit (Applied Biosystems, Branchburg, NJ, USA) according to the manufacturer’s instructions, the total RNA was then used as a template to generate first-strand cDNA for PCR amplification. The RNA and cDNA samples obtained above were used for the PCR amplification of the enterocin A, enterocin B, and enterocin P genes with the oligonucleotides listed in Table 1. The constitutively expressed 16S rRNA (63F/1387R) served as an internal control gene for these RT-PCR experiments.

The following PCR conditions were used: denaturation at 95 °C for 5 min; 35 cycles of denaturation at 94 °C for 15 s, annealing at 57 °C for 30 s, and extension at 72 °C for 60 s; and a final extension cycle at 72 °C for 5 min. The amplification products were resolved by electrophoresis in 2% agarose gels. To confirm the absence of contaminating DNA, similar experiments were conducted without reverse transcriptase.

### 2.6. Overlay Agar Spot Assay

*Enterococcus* strains with enterocin genes were grown overnight in MRS broth (Condalab) at 37 °C under aerobic conditions. Two microliters of inoculums was spot inoculated onto the MRS agar plates and grown for 18 h at 37 °C under aerobic conditions. The MRS agar plates containing the growth of *Enterococcus* strains in spot form were then overlaid with 0.75% brain–heart infusion agar (Condalab) inoculated with 10^6^ log cfu/mL of the different *L. monocytogenes* strains, and incubated at 37 °C for 24 h. Two independent trials were performed and each pathogenic strain was assayed in duplicate. The diameter of inhibition were measured and expressed in mm as the mean of *n* = 4.

## 3. Results

### 3.1. Antibiotic Susceptibility of L. monocytogenes

Zones of inhibition were measured in opened plates and with reflected light and were interpreted according the EUCAST criteria. The breakpoints of *Staphylococcus* spp. resistance were considered if no resistance criteria exist in the EUCAST or Clinical and Laboratory Standards Institute (CLSI) guidelines for *Listeria* susceptibility testing. Based on the results, the strains were classified as sensitive, intermediate resistant, or resistant.

**Figure 1:**
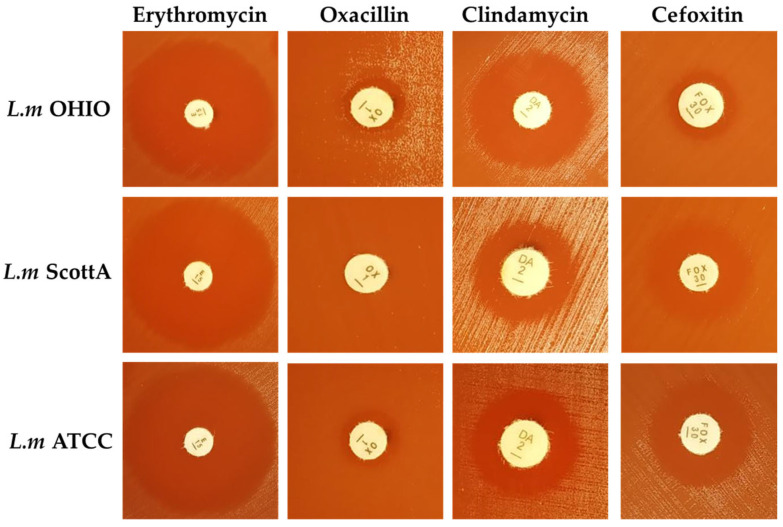
Antibiotic susceptibility testing of *L.m* OHIO, *L. monocytogenes* OHIO; *L.m* ScottA, *L. monocytogenes* Scott A; *L.m* ATCC, *L. monocytogenes* ATCC 19115 by the disk diffusion method. Erythromycin (15 µg/disc); oxacillin (1 µg/disc); clindamycin (2 µg/disc); and cefoxitin (30 µg/disc).

Fifteen antibiotics belonging to penicillins, aminoglycosides, phenicols, glycopeptides, tetracyclines, fluoroquinolones, lincosamides, macrolides, cephalosporins, sulfonamides, and carbapenems were tested. The three strains of virulent *L. monocytogenes* were sensitive to most antibiotics tested, specifically to penicillins (penicillin G, ampicillin, and amoxicillin/clavulanic acid), aminoglycosides (gentamycin), phenicols (chloramphenicol), glycopeptides (vancomycin), tetracyclines (tetracycline), fluoroquinolones (ciprofloxacin), macrolides (erythromycin), sulfonamides (trimethoprim-sulphamethoxazole), carbapenems (meropenem), and rifamycins (rifampicin), with inhibition halos between 23 mm and 39 mm (Figure 1 shows the inhibition halos corresponding to erythromycin as an example of sensitivity to the antibiotic concentrations studied).

The three strains studied exhibited resistance to oxacillin, with halos lower than 10 mm, and to clindamycin, with halos lower than 14 mm for *L. monocytogenes* Scott A and *L. monocytogenes* ATCC 19115, and between 18 and 19 mm (intermediate resistance) for *L. monocytogenes* OHIO. Moreover, *L. monocytogenes* showed intrinsic resistance to cephalosporin antibiotics (cefoxitin), with halos between 12 and 18 mm (Figure 1).

### 3.2. Detection of Enterocin Genes

A total of 41 enterococci (26 *E. faecalis* and 15 *E. faecium*) were examined for the presence of enterocin genes by means of PCR. The detected enterocins are listed in Table 2. Only *E. faecalis* INIA P290 showed the presence of the enterocin gene AS-48, whereas 10 out of 15 *E. faecium* isolates harbored one (Ent A or Ent P) or two (Ent A + Ent B or Ent P + Ent P) enterocin-encoding genes (Table 2).

### 3.3. Antimicrobial Activity

A microtiter plate assay, using strains of *L. monocytogenes* as an indicator organism, showed that only *E. faecalis* INIA P290 (Ent AS-48), *E. faecium* P442 (Ent A and Ent B), *E. faecium* P445 (Ent A and Ent B), and *E. faecium* P545 (Ent B and Ent P) were able to inhibit the growth of *L. monocytogenes* strains. The highest EU/mL was produced by *E. faecium* P545 which produced enterocin B and enterocin P (Table 2).

On the other hand, L. rhamnosus LGG, L. reuteri Biogaia, L. reuteri INIA P572, Lb. paracasei INIA P272, L. rhamnosus INIA P344, Bifidobacterium animalis BB12, Bifidobacterium pseudolongum INIA P2, and Bifidobacterium breve INIA P18 did not exhibit inhibition for any of the enterocins produced individually or in combination. Only Bifidobacterium longum BB536 showed inhibition by enterocin AS-48 and the joint production of enterocins A and B or B and P.

### 3.4. Transcriptional Analysis of Enterocins

**Figure 2:**
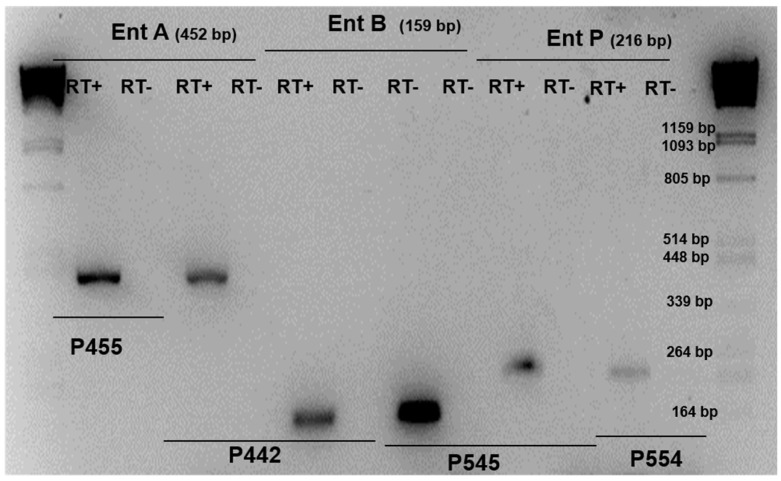
RT-PCRs were performed with total RNAs and primer sets allowing amplification of enterocin gene (lanes of RT+). To confirm the absence of contaminating DNA, similar experiments were conducted without reverse transcriptase (lanes of RT-) Control PCRs were carried out with the same primer sets and genomic DNA instead of cDNAs as template.

In some cases, enterocin genes were detected by PCR; however, we did not observe the inhibition of indicator microorganisms (Table 2), thus RT-PCR was carried out to check the expression of these genes (Figure 2).

RT-PCR analysis showed that enterocin A and enterocin B cDNA were amplified from *E. faecium* INIA P442 and *E. faecium* INA P445, and enterocin B and enterocin P cDNA were amplified from the strain *E. faecium* INIA P545, the expression of B being higher in both cases. Enterocin AS-48 cDNA was amplified from the strain *E. faecalis* INIA P290. In all these cases, the expression of the genes was correlated with the inhibition of *L. monocytogenes* strains. On the other hand, *E. faecium* INIA P553, P554, and P555 showed the expression of enterocin P; however, they did not show the inhibition of *L. monocytogenes* strains, and *E. faecium* INIA P125 and *E. faecium* INIA P552 did not show expression of enterocin A nor the inhibition of *L. monocytogenes* strains. Finally, *E. faecium* INA P454 showed the expression of enterocin A, but did not show expression of enterocin B or the inhibition of any *L. monocytogenes* strains (Table 2 and Figure 2).

### 3.5. Overlay Agar Spot Assay

**Figure 3:**
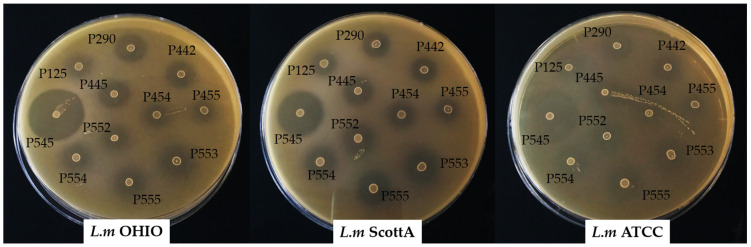
Overlay agar spot assay carry out with *Enterococcus* strains that showed the presence the enterocin genes using *L.m* OHIO, *L. monocytogenes* OHIO; *L.m* ScottA, *L. monocytogenes* Scott A; and *L.m* ATCC, *L. monocytogenes* ATCC 19115 as indicator strains.

All enterococci assayed showed a clear inhibitory antimicrobial activity around the spot against the three strains of *L. monocytogenes* (Figure 3). The enterocin B and enterocin P-producing *E. faecium* INIA P545 showed the highest diameters of inhibition (between 32 and 37 mm). The enterocin AS-48-producing *E. faecalis* INIA P290 and enterocin A and B-producing strains *E. faecium* P442 and *E. faecium* P445 resulted in values between 17 and 22 mm. The enterocin P-producing strains *E. faecium* P553, *E. faecium* P554, and *E. faecium* P555 showed diameters of inhibition between 16 and 21 mm. The lowest measurements were obtained for the enterocin A-producing or non-enterocin-producing strains *E. faecium* INIA P125, *E. faecium* INIA P454, *E. faecium* INIA P455, and *E. faecium* INIA P552 (between 10 and 18 mm).

## 4. Discussion

Unexpectedly, *L. monocytogenes* isolated from food, clinical, or environmental sources maintains a high susceptibility to a wide range of antibiotics used to treat human and animal infections except cephalosporins, fosfomycin, oxacillin, and licosamides [3]. However, *L. monocytogenes* can acquire antibiotic resistance through different mobile genetic elements from other microorganisms [38,39]. Antibiotic-resistant and multi-resistant strains of *L. monocytogenes* were first isolated in the 1980s [40].

Here, resistance to oxacillin, clindamycin, and cefoxitin in human strains of virulent *L. monocytogenes* was reported. A high variability in the percentage of resistance to oxacillin or clindamycin for *L. monocytogenes* isolates has been described in the literature [3,41]. This pathogen is usually susceptible to penicillins except for oxacillin. The *Listeria* penicillin-binding protein PBP3 is the main target of ampicillin, and this antibiotic inhibits practically all *L. mocytogenes* isolates. However, PBP3 is not inhibited by cephalosporins, being considering the cause of such intrinsic resistance [42]. Whether the mechanisms of resistance to oxacillin are related to a PBP is still unknown.

*L. monocytogenes* can also show resistance to disinfectants and heavy metals. Benzalkonium chloride resistance is associated with the overexpression of the chromosomal efflux pump MdrL, which can also extrude some kinds of antibiotics [43]. The continued use of antibiotics in the agri-food and clinical industries may lead to an increase in antibiotic resistance in *L. monocytogenes* in the near future. Therefore, we have to be prepared for an increase in the prevalence of multidrug-resistant strains of *L. monocytogenes* capable of spreading in nature.

*E. faecalis* and *E. faecium* were the predominant species of *Enterococcus* strains found in breast-fed infants isolates [18]. The frequency of enterocin genes among our isolates was higher in *E. faecium* than in *E. faecalis*, which is in accordance with previous studies [44,45]. As in this study, enterocin A and enterocin B genes are generally found to be associated [44,46,47]. The coproduction of enterocins A and B may be due to the two enterocins being secreted by one set of transport proteins, which is also related to the synergistic activities between the two bacteriocins [48,49].

The antimicrobial activity against *L. monocytogenes* observed was dependent on the presence of single bactericin genes (AS-48) or the presence of combinations of bacteriocin genes (A and B or B and P). However, the occurrence of multiple enterocin genes does not mean that the genes were expressed and secreted at the same time, as was demonstrated in the case of *E. faecium* INIA P454 (Table 2). According with our data, if two enterocins were secreted in the same supernatant, their antimicrobial activity is higher, as has been previously reported [48]. As far as we know, this is the first study in which the inhibition of *L. monocytogenes* strains was correlated with the expression of enterocin genes under the conditions tested and not with the presence of these genes. Overlay agar spot assay data are in concordance with the results with neutralized supernatants. The highest diameter of inhibition was produced by *E. faecium* P545 which simultaneously produced enterocin B and enterocin P. Acid could be responsible of the inhibition observed in the non-enterocin-producing strains and of the previous absence of inhibition observed in strains able to produce enterocin P or enterocin A individually, since their activity are usually higher at a low pH [50].

Enterocins have exhibited activity against different foodborne pathogens in different studies [51,52,53,54,55]; however, there is not much information about their effect against potential beneficial bacteria. In this work, no lactobacilli was inhibited, and *B. longum* BB536 was the only bifidobacteria sensible to the enterocins produced by the *Enterococcus* isolated from breast-fed infants. The use of bacteriocins may have an advantage compared with conventional antibiotics, since they have no destructive effect on commensal bacteria [56,57]. Treatment with antibiotics is one of the most common in childhood. Although they have no apparent adverse effects, it has been reported that antibiotics can lead to changes in gut microbiota, which can have an effect in the normal maturation of the microbiota [58]. In fact, the use of antibiotics has been associated with a reduced number of bacteria considered beneficial such as the short-chain fatty acids producers *Bifidobacteria* and *Lactobacilli*. Exposure to antibiotics has been linked with several conditions (gastrointestinal, immunologic, and neurocognitive) [59]. Moreover, it is expected that given the current use of antibiotics, this problem may even increase in the future.

The bacteriocinogenic enterococci strains are potential candidates for multiple health applications for their attractive properties such as antimicrobial compounds production and potential adaptation to different environments, including the gastrointestinal tract of humans. Although the safety of enterococci is a concern that must be taken into account, they are used as starter cultures, adjunct starters, or protective cultures, as well as probiotics for therapeutic treatments, without reported adverse effects [60,61,62]. The purified or partially purified enterocins showed in this paper can be considered target specific, safe, heat-stable, and able to exert a synergistic effect with antibiotics, which are interesting features for the development of alternative treatments against antibiotic-resistant bacterial infections without any particular safety concern. In this sense, it seems clear that the coproduction of bacteriocin can help the producer strain to avoid the appearance of resistance in some target strains [63]. The use of broad-spectrum antibiotics alters the host microbiota and therefore its functional capacity, which can have negative effects such as altered metabolic activity and the selection of antibiotic-resistant organisms [58]. Exposure to broad-spectrum antibiotics is particularly unfavorable during infancy and early childhood, since the microbiota lacks diversity and stability, making it more sensitive to environmental incursions. This can be particularly important in the development and education of the host immune system [64]. The use of bacteriocins could avoid these problems because of their target cell specificity.

On the other hand, it has been shown that some *Enterococcus* strains could reduce the infection of *L. monocytogenes* [65,66,67,68]. The use of probiotic bacteria isolated from breast-fed infants represents a valuable strategy to reduce the risk of disease due to their prophylactic and therapeutic potential [69]. The potential health risk linked to enterococcal strains must be carefully evaluated prior to any application. No virulence determinants or hemolysin activity was detected in any of the *E. faecium* studied. The gelatinase gene, when present, was silent in *E. faecium*, and none of the isolates were resistant to vancomycin. These traits together with their antilisterial activity and the presence of genes linked to colonization [18] could contribute for their use as probiotics able to exert a protective effect against bacterial infections.

## Figures and Tables

**Table 1:** Primers used in this study for enterocin-encoding gene detection.

Enterocin	Primers	Sequence (5’ to 3’)	Product Size (pb)	References
EJ97	E21-4	GCAGCTAAGCTAACGACT	279	[30]
	E21-9	AGGGGAATTTGAACAGA		
A	P9	GAGATTTATCTCCATAATCT	452	[31]
	P10	GTACCACTCATAGTGGAA		
B	EntB(f)	GAAAATGATCACAGAATGCCTA	159	[32]
	EntB(r)	GTTGCATTTAGAGTATACATTTG		
P	EntP1	ATGAGAAAAAAATTATTTAGTTT	216	[33]
	EntP2	TTAATGTCCCATACCTGCCAAACC		
MR10A	LICIJ1	ATGGGAGCAATCGCAAAA	135	[34]
	LICJ2A	TTAAATATGTTTTTTAATCCA		
L50	L50F	ATGGGAGCAATCGCAAAATTAG	98	[35]
	L50R	ATTGCCCATCCTTCTCCAAT		
AS-48	As48-1	AATAAACTACATGGGT	377	[36]
	As-48-5	CCAAGCAATAACTGCTCTTT		

**Table 2:** Enterocin-encoding genes detected by PCR in enterococci isolated from breast-fed infants feces, their gene expression, and antimicrobial activity against *Listeria monocytogenes*. The titer (expressed in enterocin units [EU] per milliliter) was defined as the reciprocal of the highest dilution causing a clear zone of inhibition.

Strain	Enterocin	Gene ^1^	Inhibition EU/mL ^2^		
		Expression	*L.m* OHIO	*L.m* ScottA	*L.m* ATCC
*E. faecium* INIA P125	A	A (-)	-	-	-
*E. faecalis* INIA P290	AS-48	AS-48 (++)	2560	1280	2560
*E. faecium* INIA P442	A, B	A (++), B (+++)	5120	2560	5120
*E. faecium* INIA P445	A, B	A (+), B (++)	2560	1280	2560
*E. faecium* INIA P454	A, B	A (+), B (-)	-	-	-
*E. faecium* INIA P455	A	A (++)	-	-	-
*E. faecium* INIA P545	B, P	B (++++), P (+)	81,920	10,240	81,920
*E. faecium* INIA P552	A	A (-)	-	-	-
*E. faecium* INIA P553	P	P (++)	-	-	-
*E. faecium* INIA P554	P	P (+)	-	-	-
*E. faecium* INIA P555	P	P (+)	-	-	-

^1^ (–) no expression, + low expression, (++) moderate expression, (+++) high expression, (++++) very high expression. ^2^ *L.m* OHIO, *L. monocytogenes* OHIO; *L.m* ScottA, *L. monocytogenes* Scott A; and *L.m* ATCC, *L. monocytogenes* ATCC 19115 as indicator strains.

## Data Availability

The data presented in this study are available on request from the corresponding author.
